# Differentiating *Iconella* from *Surirella* (Bacillariophyceae): typifying four Ehrenberg names and a preliminary checklist of the African taxa

**DOI:** 10.3897/phytokeys.82.13542

**Published:** 2017-07-03

**Authors:** Regine Jahn, Wolf-Henning Kusber, Christine Cocquyt

**Affiliations:** 1 Botanischer Garten und Botanisches Museum Dahlem, Freie Universität Berlin, Königin-Luise-Str. 6-8, 14195 Berlin, Germany; 2 Botanic Garden Meise, Nieuwelaan 38, 1680, Meise, Belgium

**Keywords:** diatoms, Surirellales, types, nomenclatural changes, biodiversity

## Abstract

To comply with the new phylogeny within the Surirellales as supported by molecular and morphological data, re-evaluations and re-combinations of taxa from and within the genera *Surirella*, *Cymatopleura*, and *Stenopterobia* and with the re-established genus *Iconella* are necessary. Since the African diatom flora is rich with taxa from these genera, especially *Iconella*, and the authors have studied these taxa recently, describing also new taxa, a preliminary checklist of African *Iconella* and *Surirella* is here presented. 94 names are contained on this list. 57 taxa have been transferred to *Iconella*; 55 taxa were formerly ranked within *Surirella* and two taxa within *Stenopterobia*. 10 taxa have stayed within *Surirella* and six taxa have been transferred from *Cymatopleura* to *Surirella*. 20 *Surirella* and 1 *Stenopterobia* names are listed which are either unrevised or unrevisable since morphological data is missing. Four names and taxa described by Ehrenberg are here typified. Two had been transferred to *Iconella* already: *Iconella bifrons* (Ehrenb.) Ruck & Nakov and *Iconella splendida* (Ehrenb.) Ruck & Nakov. Two are re-transferred from *Cymatopleura* to *Surirella*: *Surirella librile* (Ehrenb.) Ehrenb. and *Surirella undulata* (Ehrenb.) Ehrenb.; both taxa are currently known by their younger synonyms: *Cymatopleura solea* (Bréb.) W. Smith and *Cymatopleura elliptica* (Bréb. ex Kützing) W. Smith. Lectotypes for *Iconella bifrons*, *I. splendida*, *Surirella librile*, and *S. undulata* were designated.

## Introduction

*Surirella* taxa have been recognized, drawn, and described very early in diatom history since they often have large cells. The genus *Surirella* Bory is within the first published diatom genera which are still in current use: *Bacillaria* by Gmelin in 1791, *Fragilaria* by Lyngbye in 1819, *Achnanthes* and *Navicula* by Bory in 1822, *Diatoma* by Bory in 1824, *Melosira* and *Meridion* by Agardh 1824, *Surirella* by Turpin in 1828; further important genera were later described such as *Cymbella* by C. Agardh in 1830, *Gomphonema* by Ehrenberg in 1832, *Encyonema* by Kützing in 1833, *Eunotia* by Ehrenberg in 1837, *Achnanthidium* by Kützing in 1844, *Campylodiscus* by Ehrenberg ex Kützing in 1844, *Nitzschia* by Hassall in 1845, etc.

The genus name *Surirella* was introduced by P.J.F. Turpin in 1828 who had found it in a collection by the French medical doctor Suriray from brackish waters at the coast of Le Havre in France. He published beautiful drawings which had been enlarged in the microscope by 300×. Ehrenberg also used this 300x enlargement for his research and used this genus name first in 1834 for *Surirella bifrons* and *Surirella splendida*; in his 1838 publication (Ehrenberg 1838) he ranked *Surirella* as a subgenus of *Navicula* and contained in it the species *librile*, *splendida*, *bifrons*, *undulata*, *striatula* (type of the name of the genus *Surirella* introduced by Turpin), and *constricta* (no *Surirella* according to Jahn and Kusber 2004). For each of these he added a ? between the genus and the epithet which meant that he thought that this species might belong to a new genus to be differentiated from *Navicula*; at the end of the text he wrote that they definitely belong to the genus *Surirella* because of their different mode of division in comparison to *Navicula*. By 1845 Ehrenberg (1845a, b) had also recombined *Navicula librile* and *Navicula undulata* with *Surirella* (see typifications below).

Subsequently, more *Surirella* taxa were discovered. W. Smith (1851: 7) explains the morphology of *Surirella*: “Valves concave, with a longitudinal central line and margins produced beyond the suture (winged). … The concavity of the valves, their winged margins, and the longitudinal central line, which wants the central depression so conspicuous in the Naviculae, are characters which sufficiently distinguish *Surirella* from all other genera. I believe a careful examination of the loricae … would detect the presence of alae in all the species.” In this paper he also described and differentiated his new genus *Cymatopleura* against *Surirella*, the main differences being “the undulated surface of the valves seems to indicate a peculiarity of structure sufficient to constitute a generic difference, and the absence of alae and costae implies a further diversity in the internal character which cannot be regarded as unimportant” (W. Smith 1851: 12). Subsequently, W. Smith recombined *Cymatopleura solea* (= *S. librile* Ehrenb.) and *Cymatopleura elliptica* (= *S. undulata* Ehrenb.). In his *Treatise on the Diatoms*, Van Heurck (1896: 374) reintroduced and validated the genus *Stenopterobia* which had been first mentioned by Brébisson; his short differential diagnosis against *Surirella* is: “Frustules very elongated and very narrow, sometimes sigmoid.”

All the above mentioned genera, *Surirella*, *Cymatopleura*, *Stenopterobia*, *Campylodiscus* (for *C. clypeus* (Ehrenb.) Ehrenb. ex Kütz. see Poulíčková and Jahn 2007) are part of the order Surirellales (sensu Round et al. 1990, Ruck and Kociolek 2004, and Ruck et al. 2016a) which are canal-raphe-bearing diatoms with a circumferential raphe at the entire valve margin. The genera *Epithemia* and *Rhopalodia* which have a canal-raphe-not positioned around the entire valve margin, had been placed into the order Rhopalodiales (Round et al. 1990) but Ruck et al. (2016a) placed them also into the order Surirellales because their monophyly is strongly supported by molecular data (Ruck and Theriot 2011, Ruck et al. 2016a). However, the publications of Ruck et al. (2016a, 2016b), performed with morphology and molecular markers on those Surirellales, strongly reject the monophyly of several genera in the current classification (Round et al. 1990), especially concerning the genera *Surirella* and *Campylodiscus*. In order to provide a home to taxa which do not fit into their strict genus definition, Ruck et al. (2016b) reintroduced the genus *Iconella* which had been established by Jurilij in 1949 and Coronia which had been established as a subgenus by Ehrenberg, validated by Grunow and raised to genus rank by Ruck and Guiry (2016).

In the tropical African aquatic ecosystems, taxa from the genera *Surirella* and *Cymatopleura*, as traditionally known, play an important role (Ross 1983, Cocquyt and Vyverman 1994, Cocquyt 2000). In typifying historical material from African waters as described by Otto Müller (Cocquyt and Jahn 2005, 2007a, 2007b, 2007c, 2007d, 2014), by Cholnoky (Cocquyt et al. 2017), by Foged (Cocquyt and Kusber 2010), by Woodhead and Tweed (Cocquyt et al 2013), we have tried to reevaluate earlier findings of these taxa as well as their endemism. In order to help researchers to name their taxa correctly, we are providing a list of African taxa which have been recombined with a different genus; we are also listing those taxa whose names did not change. Since some of Ehrenberg’s species have been the basis for varieties of African taxa, we are including the typification of four taxa originally described by Ehrenberg and synonymizing two younger taxa.

## Material and methods

From the Ehrenberg Collection at BHUPM (Museum für Naturkunde, Berlin), the following materials (for details of the collection see Jahn and Kusber 2004) were investigated:

540128-6 (*Iconella bifrons*)

540178-1 (*Iconella splendida*)

540177-3 (*Surirella librile*)

540177-4 (*Surirella librile*)

540138-6 (*Surirella undulata*)

Zeichenblatt No 1130 (*Iconella bifrons*)

Zeichenblatt No 1160 (*Iconella splendida*)

Zeichenblatt No 1151 (*Surirella librile*)

Zeichenblatt No 1163 (*Surirella undulata*)

New names and typifications are registered in PhycoBank (Kusber et al. 2017), a registration system for nomenclatural acts (see Barkworth et al. 2016) which is currently in the trial phase. Stable http identifiers are linking to the prototype portal. When possible, we are using long-term stable and semantic web compatible identifiers for specimens according to Güntsch et al. (2017).

Two specimens at BR (Botanic Garden Meise) have been reinvestigated and documented. For specimens not seen at BRM (Alfred-Wegener-Institut für Polar- und Meeresforschung, Hustedt Diatom Study Centre, Bremerhaven), Simonsen (1987) was consulted. Author names are standardized according to IPNI database (The International Plant Names Index 2017). For several nomenclatural details the Index Nominum Algarum (1988+) and the AlgaTerra database (Jahn and Kusber 2005+) have been used.

## Results and discussion

### Typification of species described by Ehrenberg

#### Iconella bifrons

Taxon classificationPlantaeSurirellalesSurirellaceae

(Ehrenb.) Ruck & Nakov in Notulae algarum 10: 1. 2016.

≡ Navicula bifrons Ehrenb. in Abh. Königl. Akad. Wiss. Berlin 1833: 259. 1834.
≡ Surirella bifrons (Ehrenb.) Ehrenb. in Abh. Königl. Akad. Wiss. Berlin 1841: 388. 1843.

##### Lectotype

**Figure 1.**
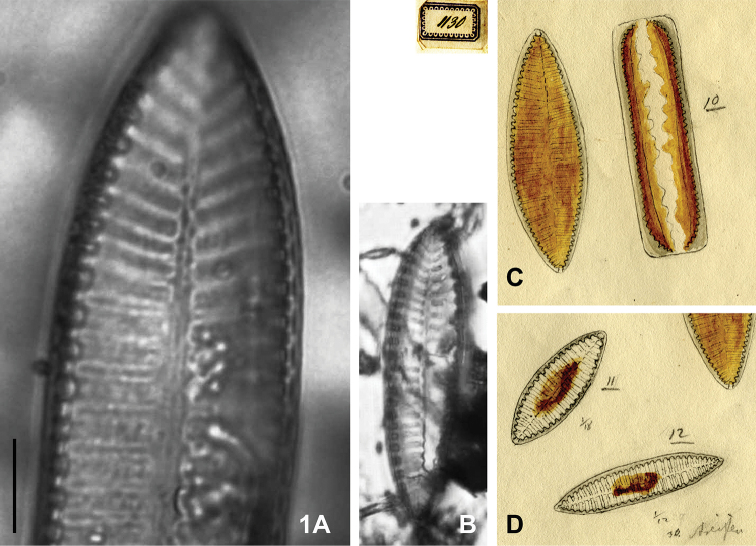
*Iconella bifrons* **A–B** Lectotype: BHUPM 540128-6 **C–D** Ehrenberg's drawing BHUPM 1130 showing different shapes of the same species in Ehrenberg's concept. Scale bar for **A** = 10 µm.

(designated here). BHUPM 540128-6 “Trockenpräparate CXXVIII 6”. (The valve representing the lectotype is reproduced here as Fig. 1A).

http://phycobank.org/100029

##### Comments.

The combination in Ehrenberg (1843) has been accepted by Kützing (1844: 61). The specimen of the lectotype was misprinted as “547806-3” in Cocquyt and Jahn (2007b) (McNeill et al. 2012, Art. 7.10). *Iconella bifrons* was introduced in Ruck et al. (2016a) and validated in Ruck et al. (2016b).

#### Iconella splendida

Taxon classificationPlantaeSurirellalesSurirellaceae

(Ehrenb.) Ruck & Nakov in Notulae algarum 10: 2. 2016.

≡ Navicula splendida Ehrenb. in Abh. Königl. Akad. Wiss. Berlin 1831: 81. 1832.
≡ Surirella splendida (Ehrenb.) Ehrenb. in Abh. Königl. Akad. Wiss. Berlin 1841: 389. 1843.

##### Lectotype

**Figure 2.**
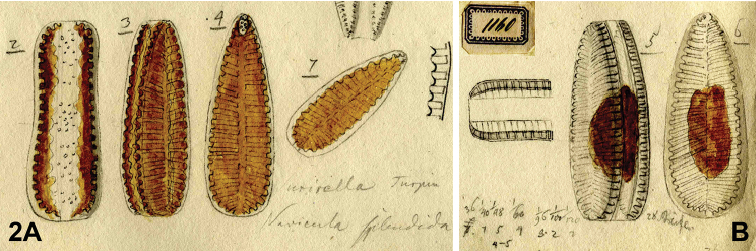
*Iconella splendida*. Lectotype: Drawing BHUPM 1160. **A** The alive cell representing the lectotype in three views (hand written numbers 2-4), length 188 µm **B** Later documentation of valve details by Ehrenberg, hand written numbers 5-6 represent a 226 µm long cell.

(designated here). [icon!] Drawing BHUPM 1160. (The cell representing the lectotype is reproduced here as Fig. 2A “2-4”).

http://phycobank.org/100030

##### Further material.

Mica preparation BHUPM 540178-1 shows a girdle view with dark inclusions and is not informative for identification.

##### Comment.

*Iconella splendida* was introduced in Ruck et al. (2016a) and validated in Ruck et al. (2016b).

#### Surirella librile

Taxon classificationPlantaeSurirellalesSurirellaceae

(Ehrenb.) Ehrenb. in Ber. Bekanntm. Verh. Königl. Preuss. Akad. Wiss. Berlin 1845: 139 table. 1845.

≡ Navicula librile Ehrenb. in Abh. Königl. Akad. Wiss. Berlin 1831: 81. 1832.

##### Lectotype

**Figure 3.**
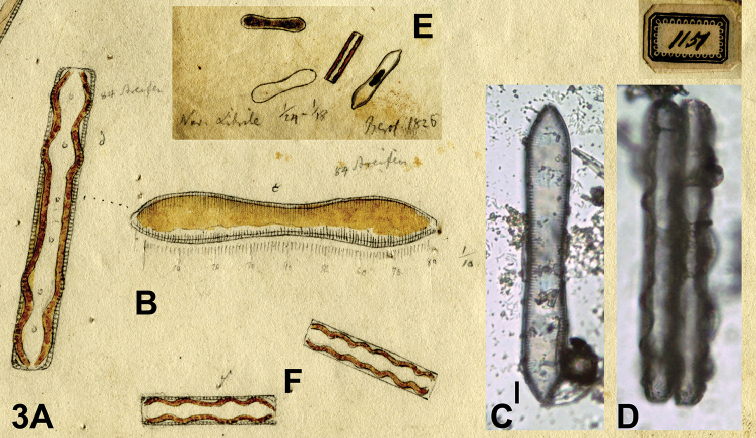
*Surirella librile*. Lectotype: Drawing BHUPM 1151. **A** Girdle view representing the lectotype (corresponding to preparation BHUPM 540177-3 in 3D) **B** Valvar view representing the lectotype; Ehrenberg indicated two views of one cell with dots between the undulated girdle view in A and the valvar view in B. **C** Corresponding preparation BHUPM 540177-4 **D** Corresponding preparation BHUPM 540177-3 **E** Documentation of Ehrenberg’s observations in 1826 **F** Small cells in girdle view not correponding to the published protologue. Scale bar for **C–D** = 10 µm.

(designated here). [icon!] BHUPM 1151c, d. (The cell representing the lectotype is reproduced here as Fig. 3A–B).

http://phycobank.org/100031

##### Further material.

BHUPM 540177-3 „Trockenpräparate CLXXVII 3“ (Fig. 3D), BHUPM 540177-4“ Trockenpräparate CLXXVII 4“ (Fig. 3C).

##### Synonyms.

= *Cymbella solea* Bréb. in Brébisson & Godey, Alg. Falaise: 51, pl. VII, p.p. 1835.

≡ *Surirella solea* (Bréb.) Bréb., Consid. Diat.: 17. 1838.

≡ *Cymatopleura solea* (Bréb.) W. Sm. in Ann. Mag. Nat. Hist. ser. 2. 7: 12. 1851.

##### Nomenclatural comment.

Ehrenberg (1845a) introduced and used the name *Surirella librile*. In this publication (1845a) he described all species new to science formally with a Latin diagnosis. Because he did not mark the species as new to science, Ehrenberg introduced the name *Surirella librile* as a new combination of *Navicula librile* under the then accepted genus name *Surirella*. This combination can be verified by the images Ehrenberg (1854) provided e.g. for Berlin material “Brakisches, strichweis lebendes, Erdlager unter Berlin” (Ehrenberg 1854: pl. 14: fig. 38).

##### Taxonomical comment.

Ehrenberg (1832) published *Navicula librile* by a description which included the length of 1/10 Paris Line which is 225.6 µm. But this measurement does not correspond to the first observations he made in Berlin 1826 drawn on a small piece of paper (Fig. 3E) and glued onto the drawing sheet BHUPM 1151. Nevertheless, the published measurement corresponds perfectly to two of his specimens on his drawing sheet BHUPM 1151 showing a living cell in valvar and girdle view (Fig. 3A–B). Therefore, Ehrenberg (1832) was the first who described the species which was some years later described again as *Cymbella solea* Bréb. & Godey (1835) which was later recombined as *Cymatopleura solea* (Bréb.) W. Sm. (1851) and became type of the name of the genus *Cymatopleura* (Smith 1851). Ehrenberg’s specimens, probably deposited in 1835 or 1836 (see Ehrenberg 1838) give proof (Fig. 3C–D) of his earlier findings (Ehrenberg 1832). In addition, Ehrenberg apparently also observed the form which is identified today as “Cymatopleura solea var. apiculata” (cf. Fig. 3F, e.g. Krammer & Lange-Bertalot 1988, Hofmann et al. 2013). Schoeman and Archibald (1979) had accepted Ehrenberg’s taxon as having priority under *Cymatopleura*. Later *Cymatopleura* was conserved against *Sphinctocystis* Hassall with *Cymatopleura solea* as its type (see Wiersema et al. 2015). Since *Cymatopleura* is here not accepted at the rank of a genus, this conservation is not applicable to our taxonomic treatment.

#### Surirella undulata

Taxon classificationPlantaeSurirellalesSurirellaceae

(Ehrenb.) Ehrenb. in Ber. Bekanntm. Verh. Königl. Preuss. Akad. Wiss. Berlin 1845: 307. 1845.

≡ Navicula? undulata Ehrenb., Infusionsthierchen, 187, pl. XXI: fig. XVI. 1838.

##### Lectotype

**Figure 4.**
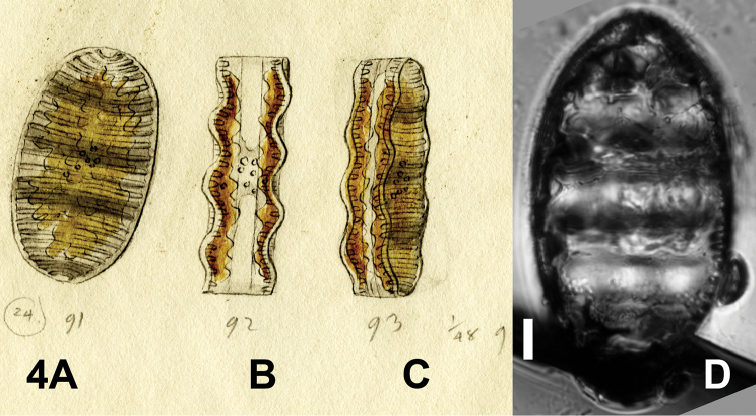
*Surirella undulata*. **A–C** Drawing BHUPM 1163 **D** Lectotype: BHUPM 540138-6, Trockenpräparate CXXXVIII 6. Scale bar for **D** = 10 µm.

(designated here). BHUPM 540138-6 “Trockenpräparate CXXXVIII 6” (The valve representing the lectotype is reproduced here as Fig. 4D).

http://phycobank.org/100032

##### Further original material.

Drawing BHUPM 1163.

**Synonyms.**

*Surirella elliptica* Bréb. ex Kütz., Kieselschal. Bacill., 61, pl. 28: fig. 28. 1844.

≡ *Cymatopleura elliptica* (Bréb. ex Kütz.) W. Sm. in Ann. Mag. Nat. Hist. ser. 2, 7: 13. 1851.

##### Comment.

Ehrenberg (1845b) introduced and used the name *Surirella undulata*. In this publication he described all species new to science formally with a Latin diagnosis. Because he did not mark the species as new to science, Ehrenberg introduced the name *Surirella undulata* as a new combination of *Navicula undulata* under the then accepted genus name *Surirella*. This combination can be verified by the drawing Ehrenberg (1854) provided e.g. for Berlin material “Brakisches, strichweis lebendes, Erdlager unter Berlin” (Ehrenberg 1854: pl. 14: fig. 39). Since Ehrenberg published this taxon name already in 1838, his name has priority over *Surirella elliptica*.

### Autapomorphies

In the Surirellaceae the raphe canal runs marginally at the edge of the valve. This canal is interrupted on the external valve face at the poles of the valve while internally the raphe is continuous at the head pole, and interrupted at the base pole. Differences between the three genera had been defined as (according to Hofmann et al 2011):

● *Cymatopleura*: valves are crossed by several large undulations which are not interrupted near the median line (= axial area). The raphe is located within a shallow keel (Spaulding and Edlund 2008).

● *Stenopterobia*: valves are elongated or curved sigmoid-like with equally sized poles. The canal raphe is raised above the valve onto a keel (Spaulding and Edlund 2010).

● *Surirella*: valves are iso- or heteropolar, transapical undulations are finely structured and interrupted near the median line.

○ *Pinnatae* group: raphe canal sits directly at the valve mantle; the raphe is interrupted at both poles. Supporting elements are the fibulae which project from the valve mantle more or less into the center of the valve face.

○ *Robustae*: raphe canal rises above valve face and mantle and is located on a wing. Where the canals of the wings, the alar canals, meet the valve face, in LM appears an apically running wavy line which has been named a loop (Schleifenbildung). Between the alar canals lie fenestrae.

These traditional differentiations based on outline, undulations and median line (formerly named pseudoraphe or axial area) were not supported by the molecular data (Ruck et al. 2016a). Ruck et al. (2016a) therefore proposed morphological autapomorphies for the differentiation of genera. As a true autapomorphy they accepted only the morphological differentiation between the *Pinnatae* and the *Robustae* group within *Surirella* which means the raphe canal is located either directly on the mantle (*Pinnatae*) or rises above the valve and mantle and has alar canals with fenestral openings occluded by fenestral bars (*Robustae*).

Since the type of the name of the genus *Surirella*, *S. striatula*, belongs to the *Pinnatae* group, the *Pinnatae* make up the true *Surirella* genus including also the taxa from the *Cymatopleura* genera because their raphe canal also is located on the valve mantle. Taxa from the *Robustae* group as well as *Stenopterobia* taxa – and a few *Campylodiscus* taxa i.e. *C. hibernicus* – belong to the reinstated genus *Iconella*. Since alar canals have also been found in marine *Campylodiscus* sensu lato (now *Coronia* (Ehrenb. ex Kütz.) Ruck & Guiry), an additional autapomorphy for *Iconella* besides the occluded fenestral openings are the internally rimmed pores.

This means that the above list of features for identifying the genera needs to be revised (according to Ruck et al. 2016):

● *Campylodiscus* s.s. (*C. clypeus* only plus formerly *Surirella Fastuosae*; most of its marine taxa are now *Coronia*, the freshwater taxa *Iconella*): communication between the raphe canal and interior through a funnel- or chalice-shaped structure.

● *Coronia* (formerly marine *Campylodiscus)*: raphe canal rises above the valve and mantle; it has alar canals with fenestral openings often unoccluded and with simple unrimmed pores.

● *Surirella* s.s. (restricted to the *SurirellaPinnatae* plus *Cymatopleura*): the raphe canal is located directly on the mantle.

● *Iconella* (formerly *SurirellaRobustae*, *Stenopterobia* plus formerly *Campylodiscus Robusti*): raphe canal rises above the valve face and mantle and has alar canals with fenestral openings occluded by fenestral bars with internally rimmed pores.

*Campylodiscus* taxa reported from tropical Africa are few. Beside the more common *C. clypeus* and C. clypeus var. bicostata (W. Sm. ex Roper) Hust. the only endemic species is *Campylodiscus tanganicae* Hust., reported from Lake Tanganyika. Since we cannot determine currently to which genus the African taxa associated historically with *Campylodiscus* belong, we have excluded them from this study. Marine *Coronia* taxa are also not part of this study.

The African *Rhopalodia* and *Epithemia* taxa as described in O. Müllers papers are currently being studied by us and will be published elsewhere.

### African *Iconella* Taxa

#### Iconella aculeata

Taxon classificationPlantaeSurirellalesSurirellaceae

(Hust.) Cocquyt & R. Jahn comb. nov.

≡ Surirella aculeata Hust. in Huber-Pestalozzi, Phytoplankt. Süsswass. vol. 2 (2), 503, fig. 609. 1942.

##### Lectotype

(designated by Simonsen 1987). BRM X1/1 Lake Tanganyika “Tanganikasee”.

http://phycobank.org/100033

- *Surirella aculeata* Hust. in A.W.F. Schmidt, Atlas Diatom.-Kunde, pl. 354: fig. 9; pl. 355: fig. 1. 1922, nom. inval.

#### Iconella acuminata

Taxon classificationPlantaeSurirellalesSurirellaceae

(Hust.) Cocquyt & R. Jahn comb. nov.

≡ Surirella acuminata Hust. in Huber-Pestalozzi, Phytoplankt. Süsswass. vol. 2 (2), 501, fig. 606. 1942.

##### Lectotype.

(designated by Simonsen 1987). BRM X1/7 Lake Tanganyika “Tanganyika See. 6”.

http://phycobank.org/100034

- *Surirella acuminata* Hust. in A.W.F. Schmidt, Atlas Diatom.-Kunde, pl. 355: 5 - 6. 1922, nom. inval.

#### Iconella anassae

Taxon classificationPlantaeSurirellalesSurirellaceae

(Cholnoky) Cocquyt & R. Jahn comb. nov.

≡ Surirella anassae Cholnoky in Oesterr. Bot. Z. 104: 84, fig. 278–279. 1957.

##### Lectotype

(designated by Cocquyt et al. 2017). UNWH NIWR 186/3707 “Tugela Village, Nkunzini”.

http://phycobank.org/100035

#### Iconella africani-orientalis

Taxon classificationPlantaeSurirellalesSurirellaceae

(Cocquyt & R. Jahn) Cocquyt & R. Jahn comb. nov.

≡ Surirella africani-orientalis Cocquyt & R. Jahn in Willdenowia 35: 364. 2005.
≡ Surirella constricta var. africana O. Müller in Bot. Jahrb. Syst. 34: 32, pl. 2: fig. 1. 1903.
≡ Surirella muelleri Hust. [non Forti] in A.W.F. Schmidt, Atlas Diatom.-Kunde, pl. 355: fig. 2 (caption). 1922, nom. illeg.

##### Lectotype

(designated by Cocquyt and Jahn 2005a). [icon] Müller 1903, pl. 2, fig. 1; reproduced as fig. 8 in Cocquyt and Jahn (2005) “Plankton of Lake Malombe [Malawi]”.

http://phycobank.org/100036

= Surirella constricta var. maxima O. Müll. in Bot. Jahrb. Syst. 34: 32, pl. 2: fig. 2. 1903.

**Lectotype** (designated by Cocquyt and Jahn 2005a). [icon] Müller 1903, pl. 2, fig. 2; reproduced as fig. 7 in Cocquyt and Jahn (2005a) “Plankton of Lake Malawi, northern part, Tanzania”.

#### Iconella agonaensis

Taxon classificationPlantaeSurirellalesSurirellaceae

(Foged) Cocquyt & R. Jahn comb. nov.

≡ Surirella agonaensis Foged in Biol. Skr. 15 (1): 123, 151, pl. 25: fig. 3. 1966.

##### Holotype.

C Ghana 141/1961. “Southwest Ghana. Fresh water (a small stream in bamboo thicket between the villages Agona and Nsuaem, Loc. No. 12). 9.III.1961.”

http://phycobank.org/100037

#### Iconella approximata

Taxon classificationPlantaeSurirellalesSurirellaceae

(Woodhead & Tweed ex Cocquyt, Jüttner & Kusber) Cocquyt, Jüttner & Kusber comb. nov.

≡ Surirella approximata Woodhead & Tweed ex Cocquyt, Jüttner & Kusber in Diatom Res. 28: 122. 2013.

##### Holotype.

NMW C90.12.179 “River Chigara, Sierra Leone”.

http://phycobank.org/100038

- *Surirella approximata* Woodhead & Tweed in Hydrobiologia 12 (2/3): 202, pl. 6 figs 71, 73. 1958, nom. inval.

#### Iconella bonsaensis

Taxon classificationPlantaeSurirellalesSurirellaceae

(Foged) Cocquyt & R. Jahn comb. nov.

≡ Surirella bonsaensis Foged in Biol. Skr. 15 (1): 124, 151, pl. 25: fig. 1. 1966.

##### Holotype.

C Ghana 151/1961. “Southwest Ghana. Fresh water (the Bonsa river, a tributary to the Ankobra river; Loc. No. 14). 9.III.1961.”

http://phycobank.org/100039

#### Iconella brevicostata

Taxon classificationPlantaeSurirellalesSurirellaceae

(O. Müll.) Cocquyt & R. Jahn comb. nov.

≡ Surirella brevicostata O. Müll. in Bot. Jahrb. Syst. 34: 34-35, pl. 2, fig. 9. 1903.

##### Lectotype

(designated by Cocquyt and Jahn 2005a). B 40 0040181 [http://herbarium.bgbm.org/object/B400040181] “Lake Malombe after discharge of Lake Nyassa [Lake Malawi, Malawi] (sample B 52 0000039 [http://herbarium.bgbm.org/object/B520000039])”.

http://phycobank.org/100040

= *Surirella tanganyikae* G.S. West in J. Linn. Soc., London. Bot. 38: 166, pl. 8: fig. 6. 1907.

##### Localities.

“Tanganyika – In plankton, Kituta Bay (25 Aug. 1904; no. 77), near Mbete (28 Sept. 1904; no. 105, and near Kala (19 Nov. 1904; no. 170)”.

#### Iconella brevicostata var. constricta

Taxon classificationPlantaeSurirellalesSurirellaceae

(Hust.) Cocquyt & R. Jahn comb. nov.

≡ Surirella brevicostata var. constricta Hust. in Huber-Pestalozzi, Phytoplankt. Süsswass. vol. 2 (2), 505, fig. 615. 1942.

##### Lectotype

(designated by Simonsen 1987). BRM 220/39 Lake Tanganyika “Tanganyika - G.S. West, Exp.”.

http://phycobank.org/100041

- Surirella brevicostata var. constricta in A.W.F. Schmidt, Atlas Diatom.-Kunde, pl. 309: fig. 2. 1914, nom. inval.

#### Iconella brevicostata var. elongata

Taxon classificationPlantaeSurirellalesSurirellaceae

(Hust. ex Simonsen) Cocquyt & R. Jahn comb. nov.

≡ Surirella brevicostata var. elongata Hust. ex Simonsen, Atlas and Catalogue of the Diatom Types of F. Hustedt 1: 50. 1987.

##### Holotype.

BRM X1/59 Lake Tanganyika “Tanganyika-See.”.

http://phycobank.org/100042

- Surirella brevicostata var. elongata Hust. in A.W.F. Schmidt, Atlas Diatom.-Kunde, pl. 309: fig. 1. 1914, nom. inval.

#### Iconella chasei

Taxon classificationPlantaeSurirellalesSurirellaceae

(Cholnoky) Cocquyt & R. Jahn comb. nov.

≡ Surirella chasei Cholnoky in Portugaliae Acta Biol. Sér. B 4: 225, fig. 118–119, 1954.

##### Lectotype

(designated by Cocquyt et al. 2017). UNWH NWU 07–172 “Eastlands, Umtali District, Southern Rhodesia (now Zimbabwe). Stream bank fully exposed to sunlight, source of mountain ravine on a fern hill on border of Eastlands”.

http://phycobank.org/100043

#### Iconella cataractarum

Taxon classificationPlantaeSurirellalesSurirellaceae

(Cocquyt & J.C. Taylor) Cocquyt & J.C. Taylor comb. nov.

≡ Stenopterobia cataractarum Cocquyt & J.C. Taylor in Phytotaxa 158: 78, figs 1–38. 2014.

##### Holotype.

BR 4345. “Zambia, Luapula Province, Ntumbachushi Falls, 09.853736° S, 28.944683° E, leg. J.C. Taylor 12-349”.

http://phycobank.org/100044

#### Iconella chepurnovii

Taxon classificationPlantaeSurirellalesSurirellaceae

(Cocquyt & R. Jahn) Cocquyt & R. Jahn comb. nov.

≡ Surirella chepurnovii Cocquyt & R. Jahn in Nova Hedwigia 84: 542, figs 45–47. 2007.

##### Holotype

(in Cocquyt and Jahn 2007d). BR 4099 (ACBUA 576) Lake Tanganyika “Lacus Tanganyika, Gatororongo (Burundi), Africa centralis”.

##### Isotype

(in Cocquyt and Jahn 2007d). B 40 0040243 [http://herbarium.bgbm.org/object/B400040243].

http://phycobank.org/100045

#### Iconella coei

Taxon classificationPlantaeSurirellalesSurirellaceae

(Cholnoky ex Cocquyt, J.C. Taylor & Kusber) Cocquyt, J.C. Taylor & Kusber comb. nov.

≡ Surirella coei Cholnoky ex Cocquyt, J.C. Taylor & Kusber in Fottea 17(1): 39, figs 30–39. 2017.

##### Holotype.

UNWH NIWR 332/6627 “Mount Kenya”.

http://phycobank.org/100046

- *Surirella coei* Cholnoky in Oesterr. Bot. Z. 107: 362, fig. 25–26, 1960, nom. inval.

#### Iconella congolensis

Taxon classificationPlantaeSurirellalesSurirellaceae

(Cocquyt & J.C. Taylor) Cocquyt & J.C. Taylor comb. nov.

≡ Surirella congolensis Cocquyt & J.C. Taylor in Eur. J. Taxon. 133: 8, figs 6–9. 2015.

##### Holotype.

BR 4399 “Oriental Province, DR Congo, Lomami River (0.49339° N and 24.16960° E). Epiphyton on dead submerged wood”.

http://phycobank.org/100047

#### Iconella crawfordii

Taxon classificationPlantaeSurirellalesSurirellaceae

(Cocquyt & R. Jahn) Cocquyt & R. Jahn comb. nov.

≡ Surirella crawfordii Cocquyt & R. Jahn in Syst. Geogr. Pl. 77: 218, fig. 3C. 2007.
≡ Surirella fuellebornii var. tumida Hust. in Huber-Pestalozzi, Phytoplankt. Süsswass. vol. 2 (2), 495, fig. 596. 1942.

##### Lectotype

(cited as holotype but in fact designated by Simonsen 1987). BRM X6/63 Lake Tanganyika “Tanganyika See”.

http://phycobank.org/100048

- Surirella fuellebornii var. tumida Hust. in A.W.F. Schmidt, Atlas Diatom.-Kunde, pl. 355: fig. 10. 1922, nom. inval.

#### Iconella debesii

Taxon classificationPlantaeSurirellalesSurirellaceae

(Hust.) Cocquyt & R. Jahn comb. nov.

≡ Surirella debesii Hust. in A.W.F. Schmidt, Atlas Diatom.-Kunde, pl. 356: figs 3, 4. 1922.

##### Lectotype

(designated by Simonsen 1987). BRM X7/59 Lake Tanganyika “Tanganikasee” of the plate.

http://phycobank.org/100049

##### Comment.

Description in the caption in Hustedt (1922).

#### Iconella delicatissima var. ghanaensis

Taxon classificationPlantaeSurirellalesSurirellaceae

(Foged) Cocquyt & Kusber comb. nov.

≡ Surirella delicatissima var. ghanaensis Foged in Biol. Skr. 15 (1): 124, 151, pl. 25: fig. 9. 1966.
≡ Stenopterobia delicatissima var. ghanensis (Foged) Cocquyt & Kusber in Nova Hedwigia 91: 126. 2010.

##### Holotype.

C Ghana 204/1961 “West Ghana. Fresh water (a small river north of the village Dwinyana; Loc. No. 30). 12.III.1961.”

http://phycobank.org/100050

- Surirella delicatissima var. africana Cholnoky 1959, nom. inval.

##### Comment.

Variety of *Iconella delicatissima* Ruck & Nakov in Notulae algarum 10: 3. 2016.

#### Iconella dodowaensis

Taxon classificationPlantaeSurirellalesSurirellaceae

(Foged) Cocquyt & R. Jahn comb. nov.

≡ Surirella dodowaensis Foged in Biol. Skr. 15 (1): 124, 151, pl. 25: fig. 6. 1966.

##### Holotype.

C Ghana 151/1961 “Southeast Ghana. Fresh water (a river near the village Dodowa, Loc. No. 62). 1.III.1961”.

http://phycobank.org/100051

#### Iconella dumae

Taxon classificationPlantaeSurirellalesSurirellaceae

(Hust.) Cocquyt & R. Jahn comb. nov.

≡ Surirella dumae Hust. in Hedwigia 63: 169. 1921.

##### Lectotype

(designated by Simonsen 1987). BRM 222/72 “D.O. Afrika. Regenpfütze im Dumagebiet” German East Africa, rain barrel.

http://phycobank.org/100052

**Figure 5.**
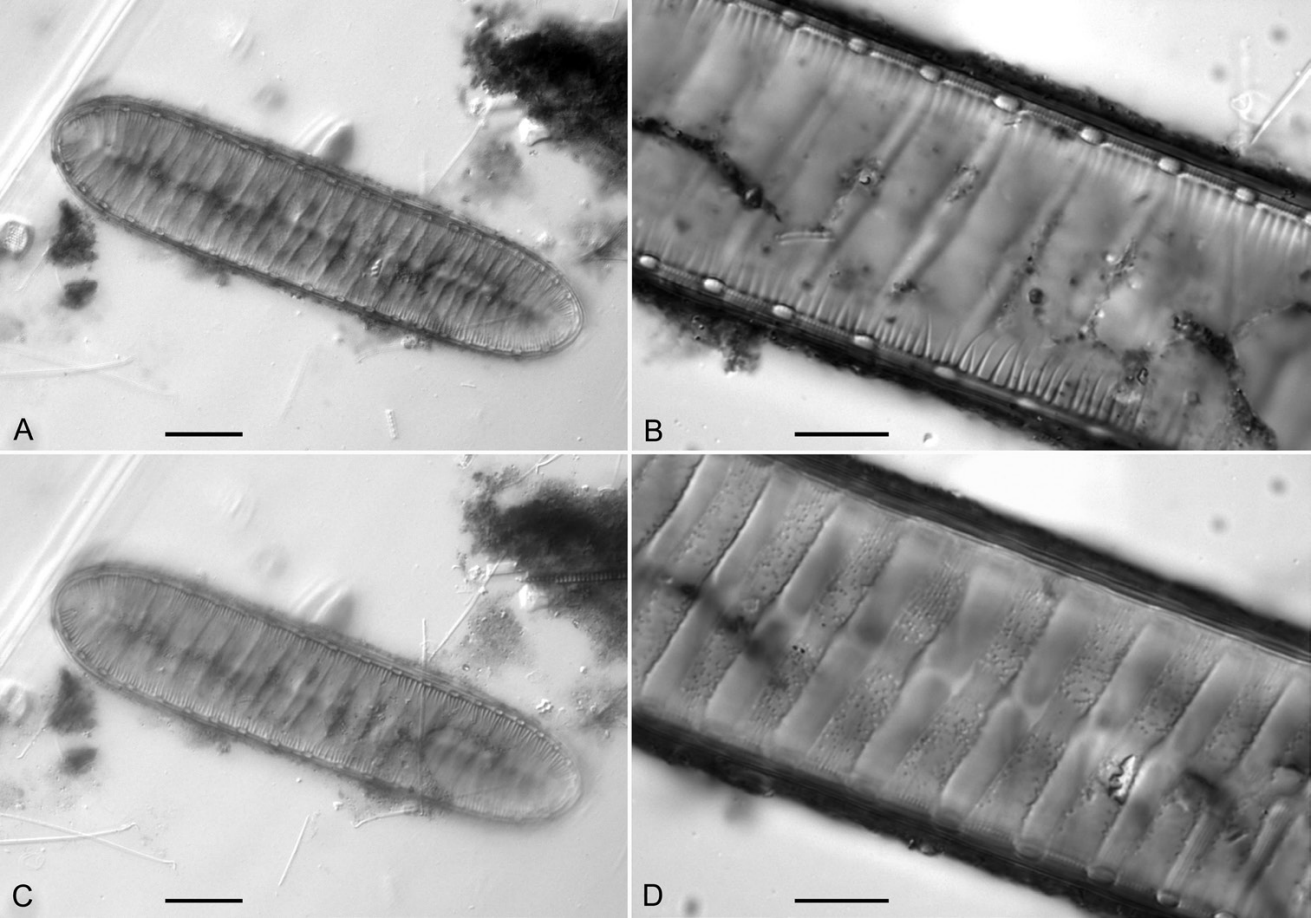
*Iconella murielae*. Valve from the holotype slide BR 984. **A, C** Overview of the entire valve at different foci **B, D** Detail of the middle part of the valve at different foci **B** is showing the fenestral openings below the raphe canal and **D** the striae and the transapical undulations. Scale bar for **A, C** = 20 µm; scale bar for **B, D** = 10 µm.

- *Surirella dumae* Hust. in A.W.F. Schmidt, Atlas Diatom.-Kunde, pl. 295: fig. 5, 6. 1913, nom. inval.

#### Iconella ebalensis

Taxon classificationPlantaeSurirellalesSurirellaceae

(Cocquyt & J.C. Taylor) Cocquyt & J.C. Taylor comb. nov.

Surirella ebalensis Cocquyt & J.C. Taylor in Eur. J. Taxon. 133: 3, figs 1–5. 2015.

##### Holotype.

BR 4398 “Oriental Province, DR Congo, Lomami River (0.49339° N and 24.16960° E). Epiphyton on *Nymphaea lotus*; collected by François Darchambeau and Ernest Tambwe on 24 Nov. 2012”.

http://phycobank.org/100053

#### Iconella effusa

Taxon classificationPlantaeSurirellalesSurirellaceae

(Hust.) Cocquyt & R. Jahn comb. nov.

≡ Surirella effusa Hust. in A.W.F. Schmidt, Atlas Diatom.-Kunde, pl. 357: figs 1, 2. 1925.

##### Lectotype

(designated by Simonsen 1987). BRM X2/9 Lake Tanganyika “Tanganika See. 6”.

http://phycobank.org/100054

##### Comment.

Although Hustedt (in Huber-Pestalozzi 1942) reported this taxon as “nicht selten” it was never observed by other investigators.

#### Iconella engleri

Taxon classificationPlantaeSurirellalesSurirellaceae

(O. Müll.) Cocquyt & R. Jahn comb. nov.

≡ Surirella engleri O. Müll. in Bot. Jahrb. Syst. 34: 28, pl. 1, fig. 4. 1903.
≡ Surirella nyassae var. engleri (O. Müll.) Ostenf. in Bull. Mus. Comp. Zool. Harvard Coll. 52: 178. 1909.

##### Lectotype

(designated by Cocquyt and Jahn 2007a). B 40 0040240 [http://herbarium.bgbm.org/object/B400040240] (the valve representing the lectotype was published as fig. 1 in Cocquyt and Jahn 2007a) “Lake Malombe after discharge of Lake Nyassa (Lake Malawi), Malawi”.

http://phycobank.org/100055

= Surirella engleri f. angustior O. Müll. in Bot. Jahrb. Syst. 34: 28, pl. 1: fig. 5. 1903.

**Lectotype** (designated by Cocquyt and Jahn 2007a). slide B 40 0040241 [http://herbarium.bgbm.org/object/B400040241] (the valve representing the lectotype was published as fig. 2 in Cocquyt and Jahn 2007a) “Lake Malombe after discharge of Lake Nyassa (Lake Malawi), Malawi”.

= Surirella engleri f. subconstricta O. Müll. in Bot. Jahrb. Syst. 34: 28-29, pl. 1, fig. 6. 1903.

**Lectotype** (designated by Cocquyt and Jahn 2007a). B 40 0040239 [http://herbarium.bgbm.org/object/B400040239] “Lake Malombe after discharge of Lake Nyassa (Lake Malawi), Malawi”.

= Surirella engleri var. constricta O. Müll. in Bot. Jahrb. Syst. 34: 29, pl. 1, figs 7, 8. 1903.

**Lectotype** (designated by Cocquyt and Jahn 2007a). B 40 0040238 [http://herbarium.bgbm.org/object/B400040238] (the valve representing the lectotype was published as fig. 4 in Cocquyt and Jahn 2007a) “Lake Malombe after discharge of Lake Nyassa (Lake Malawi), Malawi”.

= *Surirella engleri* [var. constricta] f. minor Woodhead & Tweed ex Cocquyt, Jüttner & Kusber in Diatom Res. 28:124, fig. 3. 2013.

##### Holotype.

NMW C90.12.229 “Rokupr, site E, Sierra”.

= *Surirella engleri* [var. constricta] f. sublaevis O. Müll. in Bot. Jahrb. Syst. 34: 29, pl. I, fig. 9. 1903.

**Lectotype** (designated by Cocquyt and Jahn 2007a). B 40 0040238 [http://herbarium.bgbm.org/object/B400040238] (the valve representing the lectotype was published as fig. 5 in Cocquyt and Jahn 2007a) “Lake Malombe after discharge of Lake Nyassa (Lake Malawi), Malawi”.

#### Iconella esamangensis

Taxon classificationPlantaeSurirellalesSurirellaceae

(Foged) Cocquyt & R. Jahn comb. nov.

≡ Surirella esamangensis Foged in Biol. Skr. 15 (1): 125, 151, pl. 25: fig. 2. 1966.

##### Holotype.

C Ghana 144/1961 “Southwest Ghana. Fresh water (a small river in the rain forest near the village Esamang, Loc. No. 12). 9.III.1961”.

http://phycobank.org/100056

#### Iconella friedelhinziae

Taxon classificationPlantaeSurirellalesSurirellaceae

(Cocquyt & R. Jahn) Cocquyt & R. Jahn comb. nov.

≡ Surirella friedelhinziae Cocquyt & R. Jahn in Syst. Geogr. Pl. 77: 218. 2007.
≡ Surirella fuellebornii var. elliptica O. Müll. In Bot. Jahrb. Syst. 34: 31, pl. 1: fig. 13. 1903.

##### Lectotype

(designated in Cocquyt and Jahn 2007b). [icon] Müller 1903, pl. 1, fig. 13 “Lake Tanganyika”. 1D in Cocquyt & Jahn (2007).

##### Epitype

(designated in Cocquyt and Jahn 2007b). BR 4101 “Lake Tanganyika, Burundi; near Kibwe 105 km south of Bujumbura, sandy, stony beach with abundant tufts of *Vossia cuspidata* Griff. (Poaceae)”.

##### Isoepitype

(designated in Cocquyt and Jahn 2007b). B 40 0040242 [http://herbarium.bgbm.org/object/B400040242] (ACBUA 660/2).

http://phycobank.org/100057

#### Iconella fuellebornii

Taxon classificationPlantaeSurirellalesSurirellaceae

(O. Müll.) Cocquyt & R. Jahn comb. nov.

≡ Surirella fuellebornii O. Müll. in Bot. Jahrb. Syst. 34: 30. 1903.

##### Lectotype

(designated in Cocquyt and Jahn 2007b). B 40 0040236 [http://herbarium.bgbm.org/object/B400040236] “Lake Malombe”.

http://phycobank.org/100058

= Surirella fuellebornii var. constricta O. Müll. Bot. Jahrb. Syst. 34: 30-31, pl. 1, fig. 12.1903.

**Lectotype** (designated in Cocquyt and Jahn 2007b). [icon] Müller 1903, pl. 1, fig. 12. “Lake Malombe” according to Cocquyt and Jahn (2007b).

= Surirella fuellebornii f. subconstricta O. Müll. Bot. Jahrb. Syst. 34: 30, pl. 1, fig. 11 1903.

#### Iconella gradifera

Taxon classificationPlantaeSurirellalesSurirellaceae

(Hust.) Cocquyt & R. Jahn comb. nov.

≡ Surirella gradifera Hust. in Huber-Pestalozzi, Phytoplankt. Süsswass. vol. 2 (2), 501, fig. 605. 1942.

##### Lectotype

(designated by Simonsen 1987). X2/57 Lake Tanganyika “Tanganikasee. 6”.

http://phycobank.org/100059

- *Surirella gradifera* Hust. in A.W.F. Schmidt, Atlas Diatom.-Kunde, pl. 353: fig. 8, 9. 1922, nom. inval.

#### Iconella heidenii

Taxon classificationPlantaeSurirellalesSurirellaceae

(Hust.) Cocquyt & R. Jahn comb. nov.

≡ Surirella heidenii Hust. in A.W.F. Schmidt, Atlas Diatom.-Kunde, pl. 355: fig. 2–4. 1922.

##### Lectotype

(designated by Simonsen 1987). BRM X2/58 Lake Tanganyika “Tanganyika See”.

http://phycobank.org/100060

#### Iconella kusberi

Taxon classificationPlantaeSurirellalesSurirellaceae

(Cocquyt & R. Jahn) Cocquyt & R. Jahn comb. nov.

≡ Surirella kusberi Cocquyt & R. Jahn in Syst. Geogr. Pl. 77: 221. 2007.
≡ Surirella bifrons var. intermedia O. Müll. in Bot. Jahrb. Syst. 34: 27, pl. 1: fig. 1. 1903.

##### Lectotype

(designated by Cocquyt and Jahn 2007b). [icon] Pl. 1. fig. 1 in Müller (1903) reproduced as fig. 1A in Cocquyt and Jahn (2007b) “unknown” locality.

##### Epitype

(designated by Cocquyt and Jahn 2007b). Slide B 40 0040235 [http://herbarium.bgbm.org/object/B400040235], from Müller's material B 52 0000058 [http://herbarium.bgbm.org/object/B520000058] (the valve representing the epitype in Cocquyt and Jahn 2007b as fig. 7C “The River Olunga (Tanzania)”.

http://phycobank.org/100061

#### Iconella lancettula

Taxon classificationPlantaeSurirellalesSurirellaceae

(Hust.) Cocquyt & R. Jahn comb. nov.

≡ Surirella lancettula Hust. in Huber-Pestalozzi, Phytoplankt. Süsswass. vol. 2 (2), 505, fig. 613. 1942.

##### Lectotype

(cited as holotype but in fact designated by Simonsen 1987). BRM X7/58 Lake Tanganyika “Tanganikasee”.

http://phycobank.org/100062

- *Surirella lancettula* Hust. in A.W.F. Schmidt, Atlas Diatom.-Kunde, pl. 354: figs 1, 2. 1922, nom. inval.

#### Iconella latecostata

Taxon classificationPlantaeSurirellalesSurirellaceae

(Hust.) Cocquyt & R. Jahn comb. nov.

**Figure 6.**
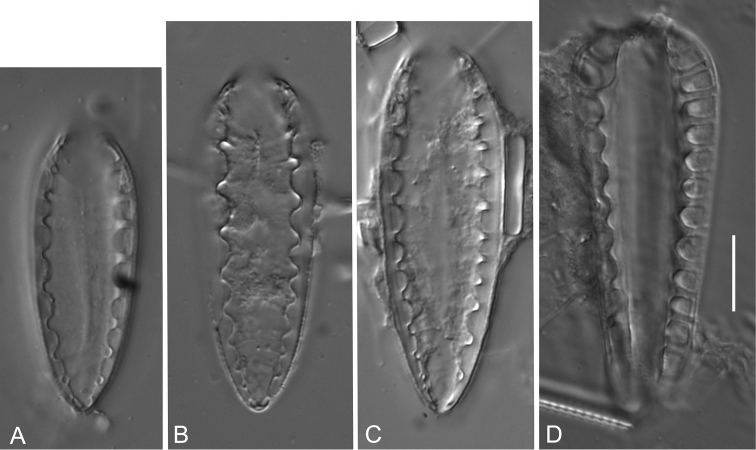
*Iconella tchadensis*. **A–D** Valves from the holotype slide BR 987 **A–C** valvar views showing the size range **D** girdle view. Scale bar = 10 µm.

≡ Surirella latecostata Hust. in A.W.F. Schmidt, Atlas Diatom.-Kunde, pl. 353: figs 5–7. 1922.

##### Lectotype

(designated by Simonsen 1987). BRM X2/70 Lake Tanganyika “Tanganyika See.”

http://phycobank.org/100063

#### Iconella likomensis

Taxon classificationPlantaeSurirellalesSurirellaceae

(Cocquyt & R. Jahn) Cocquyt & R. Jahn comb. nov.

≡ Surirella likomensis Cocquyt & R. Jahn in Willdenowia 35: 361. 2005.
≡ Surirella bifrons [var. tumida] f. minor O. Müll. in Bot. Jahrb. Syst. 34: 28, pl. 1, fig. 3. 1903.

##### Lectotype

(designated by Cocquyt and Jahn 2005a). [icon] Müller 1903: t. 1, fig. 3; reproduced as Fig. 4 in Cocquyt and Jahn (2005) Lake Nyassa [Lake Malawi] near Likoma on the bottom.

##### Epitype

(designated by Cocquyt and Jahn 2005a). B 40 0040180 [http://herbarium.bgbm.org/object/B400040180] Lake Malombe after discharge of Lake Malawi, Malawi (sample B 52 0000039 [http://herbarium.bgbm.org/object/B520000039].

http://phycobank.org/100064

##### Taxonomical remark.

According to Cocquyt and Jahn (2005a), the taxonomic concept Surirella biseriata var. bifrons (Ehrenb.) Hust. sec. Hustedt in Schmidt (1912) pro parte falls into synonymy.

#### Iconella linearis var. elliptica

Taxon classificationPlantaeSurirellalesSurirellaceae

(O. Müll.) Cocquyt & R. Jahn comb. nov.

≡ Surirella linearis var. elliptica O. Müll. in Bot. Jahrb. Syst. 34: 30, pl. 1: fig.10. 1903.

##### Lectotype

(designated by Cocquyt and Jahn 2005a). B 40 0040182 [http://herbarium.bgbm.org/object/B400040182] Lake Malombe after discharge of Lake Malawi, Malawi (sample B 52 0000039 [http://herbarium.bgbm.org/object/B520000039]).

http://phycobank.org/100065

##### Comment.

Variety of *Iconella linearis* (W. Sm.) Ruck & Nakov in Notulae algarum 10: 2. 2016.

#### Iconella linearis var. elongata

Taxon classificationPlantaeSurirellalesSurirellaceae

(Compère) Cocquyt & R. Jahn, comb. nov. et stat. nov.

Surirella linearis f. elongata Compère in Bull. Jard. Bot. Nat. Belg. 45: 380, figs 11, 23. 1975.

##### Holotype.

BR 982 “Chad, Lake Chad”.

http://phycobank.org/100066

#### Iconella malombae

Taxon classificationPlantaeSurirellalesSurirellaceae

(O. Müll.) Cocquyt & R. Jahn comb. nov.

≡ Surirella malombae O. Müll. in Bot. Jahrb. Syst. 34: 34, pl. 2: figs 5–6. 1903.
≡ Surirella nyassae var. malombae (O. Müll.) Ostenf. in Bull. Mus. Comp. Zool. Harvard Coll. 52: 178. 1909.

##### Lectotype

(designated by Cocquyt and Jahn 2007d). B 40 0040230 [http://herbarium.bgbm.org/object/B400040230] “Lake Malombe after discharge of Lake Nyasa (Lake Malawi), Malawi” (sample B 52 0000039 [http://herbarium.bgbm.org/object/B520000039])”.

= Surirella malombae f. acuta O. Müll. in Bot. Jahrb. Syst. 34: 34, pl. 2, fig. 7. 1903.

**Lectotype** (designated by Cocquyt and Jahn 2007d). [icon] Pl. 2: fig. 7 in Müller (1903) “Lake Malombe, after discharge of Lake Malawi, Malawi”.

##### Epitype

(designated by Cocquyt and Jahn 2007d). B 40 0040231 [http://herbarium.bgbm.org/object/B400040231] “Lake Victoria near the isle of Djuma” (sample B 52 0000100 [http://herbarium.bgbm.org/object/B520000100]).

http://phycobank.org/100067

= Surirella malombae var. tumida Ostenf. in Bot. Jahrb. Syst. 41: 343. 1908.

#### Iconella margaritifera

Taxon classificationPlantaeSurirellalesSurirellaceae

(Hust.) Cocquyt & R. Jahn comb. nov.

≡ Surirella margaritifera Hust. in Huber-Pestalozzi, Phytoplankt. Süsswass. vol. 2 (2), 501, fig. 607. 1942.

##### Lectotype

(designated by Simonsen 1987). BRM X2/85 Lake Tanganyika “Tanganyika See. 6”.

http://phycobank.org/100068

- *Surirella margaritifera* Hust. in A.W.F. Schmidt, Atlas Diatom.-Kunde, pl. 354: figs 3–5. 1922, nom. inval.

#### Iconella margaritacea

Taxon classificationPlantaeSurirellalesSurirellaceae

(O. Müll.) Cocquyt & R. Jahn comb. nov.

≡ Surirella margaritacea O. Müll. in Bot. Jahrb. Syst. 34: 37, pl. 2: fig. 12. 1903.

##### Lectotype

(designated by Cocquyt and Jahn 2005a). slide B 40 0040183 [http://herbarium.bgbm.org/object/B400040183], river Songwe [Tanzania] (sample B 52 0000036 [http://herbarium.bgbm.org/object/B520000036]).

http://phycobank.org/100069

#### Iconella muelleri

Taxon classificationPlantaeSurirellalesSurirellaceae

(Forti) Cocquyt & R. Jahn comb. nov.

≡ Surirella muelleri Forti in Atti R. Ist. Veneto Sc. Lett. Ed Arti 69(2): 1284, 1294, pl. 3: fig. 9, 10. 1910.

##### Type locality.

Ethiopia, lago Zulay. Coll. Giov. Negri.

http://phycobank.org/100070

#### Iconella murielae

Taxon classificationPlantaeSurirellalesSurirellaceae

(Compère) Cocquyt & R. Jahn comb. nov.

≡ Surirella murielae Compère in Bull. Jard. Bot. Nat. Belg. 45: 381, figs 12, 26. 1975.

##### Holotype.

BR 984, Compère 3875. “Lac Tchad, à 10 km au N du delta du Chari, plancton”.

http://phycobank.org/100071

##### Comment.

This species is illustrated by LM and SEM in Bogaerts et al. (2014), additional illustrations are given here in Fig. 5.

#### Iconella nagbogensis

Taxon classificationPlantaeSurirellalesSurirellaceae

(Foged) Cocquyt & R. Jahn comb. nov.

≡ Surirella nagbogensis Foged in Biol. Skr. 15 (1): 125, 151, pl. 25: fig. 7. 1966.

##### Holotype.

C Ghana 279/1961 “Northeast Ghana. Fresh water (a small river near the village Nagbog, Loc. No. 53). 21.III.1961”.

http://phycobank.org/100072

#### Iconella nervosa

Taxon classificationPlantaeSurirellalesSurirellaceae

(A.W.F. Schmidt) Cocquyt & R. Jahn comb. nov.

≡ Surirella tenera var. nervosa A.W.F. Schmidt, Atlas Diatom.-Kunde, pl. 23: fig. 15. 1875.
≡ Surirella nervosa (A.W.F. Schmidt) Ant. Mayer in Ber. Naturwiss. Vereins Regensburg 14: 341. 1913.

##### Lectotype

(here designated). [icon!] A.W.F. Schmidt, Atlas Diatom.-Kunde, pl. 23: fig. 15. “Whatabevot”

http://phycobank.org/100073

##### Taxonomical comment.

From two different localities A.W.F. Schmidt (1875) depicted three valves with an axial area including a central line and spines at both ends of this line. The valve depicted as pl. 23: fig. 15 fits the criterium “illustration with analyses” (McNeill et al. 2012, Art. 38.10) because many small spinules on the valve surface are clearly shown. Therefore we have choosen pl. 23: fig. 15 as the lectotype. fig. 16 is less detailed. We exclude the depicted specimen collected at Khayenmatay (fig. 17) from the species because with its denser costae and less distinct wing projection it probably belongs to a different species.

#### Iconella nyassae

Taxon classificationPlantaeSurirellalesSurirellaceae

(O. Müll.) Cocquyt & R. Jahn comb. nov.

≡ Surirella nyassae O. Müll. in Bot. Jahrb. Syst. 34: 33, pl. 2: fig. 3. 1903.

##### Lectotype

(designated by Cocquyt and Jahn 2007d). B 40 0040228 “Lake Malawi near Langenburg, Tanzania, between 40-70 m depth (sample B 52 000014 [http://herbarium.bgbm.org/object/B520000014]).

http://phycobank.org/100074

= Surirella nyassae var. sagitta O. Müll. in Bot. Jahrb. Syst. 34: 33, pl. 2: fig. 4. 1903.

**Lectotype** (designated by Cocquyt and Jahn 2007d). B 40 0040229 “Lake Malawi near Langenburg, Tanzania, between 40-70 m depth” (sample B 52 000013 [http://herbarium.bgbm.org/object/B520000013]).

#### Iconella obtusiuscula

Taxon classificationPlantaeSurirellalesSurirellaceae

(G.S. West) Cocquyt & R. Jahn comb. nov.

≡ Surirella obtusiuscula G.S. West in J. Linn. Soc., London Bot. 38: 165, pl. 8: fig. 7. 1907.

##### Comment.

Type specimen not studied but specimens from Lake Tanganyika observed (Cocquyt 1998).

##### Localities.

“Tanganyika – In plankton, Komba Bay (11 Oct. 1904; no. 135) and near Kala (19 Nov. 1904; no. 170).”

http://phycobank.org/100075

#### Iconella oliffii

Taxon classificationPlantaeSurirellalesSurirellaceae

(Cholnoky) Cocquyt & R. Jahn comb. nov.

≡ Surirella oliffii Cholnoky in Oesterr. Bot. Z. 103: 90, fig. 134, 1956.

##### Lectotype

(designated by Cocquyt et al. 2017). [icon]. fig. 134 in Cholnoky (1956) “Umgeni river by Albert Falls. 14.X.1954”, leg. W.D. Oliff.

##### Epitype

(designated by Cocquyt et al. 2017). UNWH NIWR 193/3860 “Kwa–Zulu Natal, Umgeni River at Albert Falls, Umgeni, South Africa”.

http://phycobank.org/100076

#### Iconella panganiensis

Taxon classificationPlantaeSurirellalesSurirellaceae

(O. Müll.) Cocquyt & R. Jahn comb. nov.

≡ Surirella panganiensis O. Müll. in Bot. Jahrb. Syst. 34: 257-258, figs 3–4. 1904.

##### Lectotype

(designated by Cocquyt and Jahn 2005). [icon] Müller 1904, fig. 3; reproduced as fig. 22 in Cocquyt and Jahn (2005a), Rufidji (Usambara-Usagara region) Pangani rapids.

http://phycobank.org/100077

#### Iconella plana

Taxon classificationPlantaeSurirellalesSurirellaceae

(G.S. West) Cocquyt & R. Jahn comb. nov.

≡ Surirella plana G.S. West in J. Linn. Soc., London Bot. 38: 165, pl. 8: fig. 5. 1907.

##### Locality.

“Tanganyika – In plankton, near Ndauvie (7 Feb. 1905; no. 227).”

http://phycobank.org/100078

##### Comment.

Type specimen not studied but specimens from Lake Tanganyika observed (Cocquyt 1998).

#### Iconella propinqua

Taxon classificationPlantaeSurirellalesSurirellaceae

(Hust.) Cocquyt & R. Jahn comb. nov.

≡ Surirella propinqua Hust. in Exploration du Parc National Albert, Mission H. Damas 8: 153, pl. 14: fig. 5, 6. 1949.

##### Lectotype

(designated by Simonsen 1987), BRM 242/6 DR Congo “Belg. Kongo. 39”.

http://phycobank.org/100079

#### Iconella pseudothienemannii

Taxon classificationPlantaeSurirellalesSurirellaceae

(Cholnoky) Cocquyt & R. Jahn comb. nov.

≡ Surirella pseudothienemannii Cholnoky in Beih. Nova Hedwigia 21: 72–73, fig. 184, 185, 1966.

##### Holotype.

UNWH NIWR 169/336 „Uferwasser des Kunene-Flusses bei Swart Boois Drift. Stille Bucht am Südufer, 8.8.1961“.

http://phycobank.org/100080

#### Iconella reicheltii

Taxon classificationPlantaeSurirellalesSurirellaceae

(Hust.) Cocquyt & R. Jahn comb. nov.

≡ Surirella reicheltii Hust. in Huber-Pestalozzi, Phytoplankt. Süsswass. vol. 2 (2), 501, fig. 607. 1942.

##### Lectotype

(designated by Simonsen 1987). BRM X3/69 Lake Tanganyika “Tanganyika See”.

http://phycobank.org/100081

- *Surirella reicheltii* Hust. in A.W.F. Schmidt, Atlas Diatom.-Kunde, pl. 354: figs 3–5. 1922, nom. inval.

#### Iconella sorriensis

Taxon classificationPlantaeSurirellalesSurirellaceae

(Foged) Cocquyt & R. Jahn comb. nov.

≡ Surirella sorriensis Foged in Biol. Skr. 15 (1): 125, 152, pl. 25: fig. 8. 1966.

##### Holotype.

C Ghana 223/1961 “North Ghana. Fresh water (the Sorri river, the White Volta river system, Loc. No. 35). 16.III.1961”.

http://phycobank.org/100082

#### Iconella spiraloides

Taxon classificationPlantaeSurirellalesSurirellaceae

(Hust.) Cocquyt & R. Jahn comb. nov.

≡ Surirella spiraloides Hust. in Huber-Pestalozzi, Phytoplankt. Süsswass. vol. 2 (2), 507, fig. 617. 1942.

##### Lectotype

(designated by Simonsen 1987). BRM X4/45 Lake Tanganyika “Tanganika See. 6”.

http://phycobank.org/100083

- *Surirella spiraloides* Hust. in A.W.F. Schmidt, Atlas Diatom.-Kunde, pl. 353: fig. 2, 3. 1922, nom. inval.

#### Iconella subcontorta

Taxon classificationPlantaeSurirellalesSurirellaceae

(Hust.) Cocquyt & R. Jahn comb. nov.

≡ Surirella subcontorta Hust. in Huber-Pestalozzi, Phytoplankt. Süsswass. vol. 2 (2), 518, fig. 633. 1942.

##### Lectotype

(designated by Simonsen 1987). BRM X4/57 Lake Tanganyika “Tanganyika See”.

http://phycobank.org/100084

- *Surirella subcontorta* Hust. in A.W.F. Schmidt, Atlas Diatom.-Kunde, pl. 356: fig. 1, 2. 1922, nom. inval.

#### Iconella takoradiensis

Taxon classificationPlantaeSurirellalesSurirellaceae

(Foged) Cocquyt & R. Jahn comb. nov.

≡ Surirella takoradiensis Foged in Biol. Skr. 15 (1): 126, 152, pl. 25: fig. 4. 1966.

##### Holotype.

C Ghana 119/1961 “Southest Ghana. Fresh water (a small river in the rain forest west of Takoradi; Loc. No. 8) 8.III.1961”.

http://phycobank.org/100085

= Surirella takoradiensis var. suhinensis Foged in Biol. Skr. 15 (1): 126, 152, pl. 25: fig. 5.

**Holotype.** C Ghana 218/1961 “West Ghana. Fresh water (the Suhin river, the Black Volta river system; Loc. No. 33. 13.III.1961”.

#### Iconella tchadensis

Taxon classificationPlantaeSurirellalesSurirellaceae

(Compère) Cocquyt & R. Jahn comb. nov.

≡ Surirella tchadensis Compère in Bull. Jard. Bot. Nat. Belg. 45: 380, figs 11, 23. 1975.

##### Holotype.

BR 987 (see also Bogaerts et al. 2014), Compère 3880, Tchad.

Valves from the holotype slide are given in Fig. 6

http://phycobank.org/100086

#### Iconella tumida

Taxon classificationPlantaeSurirellalesSurirellaceae

(O. Müll.) Cocquyt & R. Jahn comb. nov.

≡ Surirella bifrons var. tumida O. Müll. in Bot. Jahrb. Syst. 34: 27, t. 1, fig. 2. 1903.
≡ Surirella tumida (O. Müll.) Cocquyt & R. Jahn in Willdenowia 35: 361. 2005.

##### Lectotype

(designated by Cocquyt and Jahn 2005). [icon] Müller 1903: t. 1, fig. 2; reproduced as fig. 1 in Cocquyt and Jahn (2005a) “Lake Malombe after discharge of Lake Nyassa [Lake Malawi, Malawi]”.

##### Epitype

(designated by Cocquyt and Jahn 2005a). B 40 0040179 [http://herbarium.bgbm.org/object/B400040179] “Lake Malombe after discharge of Lake Malawi, Malawi (sample B 52 0000038 [http://herbarium.bgbm.org/object/B520000038])”.

http://phycobank.org/100087

##### Taxonomical remark.

According to Cocquyt and Jahn (2005a), the taxonomic concept Surirella biseriata var. bifrons (Ehrenb.) Hust. sec. Hustedt (in Schmidt 1911) pro parte falls into synonymy.

#### Iconella turbo

Taxon classificationPlantaeSurirellalesSurirellaceae

(O. Müll.) Cocquyt & R. Jahn comb. nov.

≡ Surirella turbo O. Müll. in Bot. Jahrb. Syst. 34: 34, pl. 2, fig. 8. 1903.

##### Lectotype

(designated by Cocquyt and Jahn 2005a). [icon] Müller 1903, pl. 2, fig. 8; reproduced as fig. 24 in Cocquyt and Jahn (2005a) “Lake Malawi, near Island of Likoma (sample B 52 0000025 [http://herbarium.bgbm.org/object/B520000025])”.

http://phycobank.org/100088

#### Iconella vasta

Taxon classificationPlantaeSurirellalesSurirellaceae

(Hust.) Cocquyt & R. Jahn comb. nov.

≡ Surirella vasta Hust. in Huber-Pestalozzi, Phytoplankt. Süsswass. vol. 2 (2), 503, fig. 611. 1942.

##### Lectotype

(designated by Simonsen 1987). BRM X4/89 Lake Tanganyika “Tanganyika See. 6”.

http://phycobank.org/100089

- *Surirella vasta* Hust. in A.W.F. Schmidt, Atlas Diatom.-Kunde, pl. 354: fig. 6, 7. 1922, nom. inval.

#### Iconella vasta var. linearis

Taxon classificationPlantaeSurirellalesSurirellaceae

(Hust.) Cocquyt & R. Jahn comb. nov.

≡ Surirella vasta var. linearis Hust. in Huber-Pestalozzi, Phytoplankt. Süsswass. vol. 2 (2), 504. 1942.

##### Lectotype

(cited as holotype but in fact designated by Simonsen 1987). X4/9589 Lake Tanganyika “Tanganyika See. 6”.

http://phycobank.org/100090

### Updated taxonomy of African *Surirella* taxa

#### Surirella afrocalcarata

Taxon classificationPlantaeSurirellalesSurirellaceae

Cocquyt & R. Jahn nom. nov.

≡ Cymatopleura calcarata Hust. in Huber-Pestalozzi, Phytoplankt. Süsswass. vol. 2 (2), 480, fig. 579. 1942.

##### Lectotype

(designated by Simonsen 1987). BRM Xa/20 Lake Tanganyika “Tanganika See”.

http://phycobank.org/100091

- *Cymatopleura calcarata* Hust. in A.W.F. Schmidt, Atlas Diatom.-Kunde, pl. 367: figs 1–2. 1927, nom. inval.

##### Comment.

The epithet name “afrocalcarata” was chosen because of *Suriraya calcarata* Pfitzer in Bot. Abh. Morphol. Physiol. 2: 107. 1871. *Suriraya* is a homotypic synonym of *Surirella* Turpin.

#### Surirella clavata

Taxon classificationPlantaeSurirellalesSurirellaceae

(O. Müll.) Cocquyt & R. Jahn comb. nov.

≡ Cymatopleura solea var. clavata O. Müll. in Bot. Jahrb. Syst. 34: 22, fig. 1. 1904.
≡ Cymatopleura clavata (O. Müll.) Cocquyt & R. Jahn in Pl. Ecol. Evol. 147 (3): 413 2014.

##### Lectotype

(designated by Cocquyt and Jahn 2014). B 40 0040250 [http://herbarium.bgbm.org/object/B400040250] (the valve representing the lectotype was published as fig. 1D in Cocquyt and Jahn (2014) “Malawi, Lake Malombe, after the discharge of Lake Malawi”.

http://phycobank.org/100092

#### Surirella comperei

Taxon classificationPlantaeSurirellalesSurirellaceae

(Cocquyt & R. Jahn) Cocquyt & R. Jahn comb. nov.

≡ Cymatopleura comperei Cocquyt & R. Jahn in Pl. Ecol. Evol. 147 (3): 419, figs 6–8. 2014.

##### Holotype.

B 40 0040184 [http://herbarium.bgbm.org/object/B400040184]; the valve representing the holotype was published as fig. 6E in Cocquyt and Jahn (2014) “Malawi, Lake Malawi near Langenburg”.

http://phycobank.org/100093

- Cymatopleura solea var. subconstricta O. Müll. in Bot. Jahrb. Syst. 34: 23. 1904, nom. inval.

- *Cymatopleura solea* var. [*subconstricta*] f. major O. Müll. in Bot. Jahrb. Syst. 34: 23. 1904, nom. inval.

- *Cymatopleura solea* var. [*subconstricta*] f. minor O. Müll. in Bot. Jahrb. Syst. 34: 23. 1904, nom. inval.

- *Cymatopleura solea* var. [*subconstricta*] f. minor O. Müll. in A.W.F.Schmidt, Atlas Diatom.-Kunde, pl. 245: fig. 3. 1904, nom. inval.

#### Surirella distinguenda

Taxon classificationPlantaeSurirellalesSurirellaceae

Hust. in A.W.F. Schmidt, Atlas Diatom.-Kunde, pl. 283: fig. 5. 1912.

##### Lectotype

(cited as holotype but in fact designated by Simonsen 1987). BRM 218/56.

“Togo, Westafrika 1912, Lagunenschlick”.

#### Surirella fasiculata

Taxon classificationPlantaeSurirellalesSurirellaceae

O. Müll. in Bot. Jahrb. Syst. 34: 36, pl. 1: fig. 14. 1903.

##### Lectotype

(designated by Cocquyt and Jahn 2007c). [icon] Müller (1903): pl. 1: fig. 14 “Lake Nogzi, a brackish water lake in the crater of the mountain Nogzi on the northern edge of Kondeland, at 2000 m asl, Tanzania”.

##### Epitype

(designated by Cocquyt and Jahn 2007c). B 40 0040234 [http://herbarium.bgbm.org/object/B400040234] (the valve representing the epitype was illustrated as fig. 4 in Cocquyt and Jahn 2007c) “basin near the hot spring of Utengule, Beya Mountain (Tanzania)”.

#### Surirella laticeps

Taxon classificationPlantaeSurirellalesSurirellaceae

(O. Müll.) Cocquyt & R. Jahn comb. nov.

≡ Cymatopleura solea var. laticeps O. Müll. in Bot. Jahrb. Syst. 34: 22-23, fig. 2. 1904.
≡ Cymatopleura laticeps (O. Müll.) Cocquyt & R. Jahn in Pl. Ecol. Evol. 147 (3): 418. 2014.

##### Lectotype

(designated in Cocquyt and Jahn 2014). B 40 0040251 [http://herbarium.bgbm.org/object/B400040251] (the valve representing the lectotype was published as fig. 5 B in Cocquyt and Jahn (2014) “‘Nyassaland’, Tanzania, Lake Malawi near Langenburg”.

http://phycobank.org/100094

#### Surirella modesta

Taxon classificationPlantaeSurirellalesSurirellaceae

Hust. in A.W.F. Schmidt, Atlas Diatom.-Kunde, pl. 357: fig. 8, 9. 1925.

##### Lectotype

(designated by Simonsen 1987). BRM X2/87 Cameroon “Kamerun, Lagune”.

#### Surirella nyansae

Taxon classificationPlantaeSurirellalesSurirellaceae

(G.S. West) Cocquyt & R. Jahn comb. nov.

≡ Cymatopleura nyansae G.S. West in J. Linn. Soc. Bot. 38: 167, pl. 8: fig. 8. 1907.

##### Lectotype

(designated in Cocquyt and Jahn 2014). BM 34183 “Tanganyika – In plankton, near Kala (19 Nov. 1904; no. 170).”

http://phycobank.org/100095

#### Surirella olungensis

Taxon classificationPlantaeSurirellalesSurirellaceae

Cocquyt & R. Jahn in Cryptog. Algol. 28: 111, figs 7–12, 18–21. 2007.

##### Holotype.

B 40 0040232 [http://herbarium.bgbm.org/object/B400040232] (the valve representing the holotype is illustrated as fig. 7 in Cocquyt and Jahn 2007c) “Olunga River (Ohmga) in Ussangu northern Mount Kinga, Tanzania (sample B 52 0000058 [http://herbarium.bgbm.org/object/B520000058])”.

#### Surirella ostentata

Taxon classificationPlantaeSurirellalesSurirellaceae

Cholnoky in Hydrobiologia 19: 106, 1962.

≡ Surirella ovata var. africana Cholnoky in Ber. Deutsch. Bot. Ges. 68: 21–22, fig. 46. 1955.

##### Lectotype

(designated by Cocquyt et al. 2017). UNWH NIWR 191/3802 “Bewässerungskanal bei Vredendal near Olifantsriver”, leg. A.H.P. Engelbrecht.

http://phycobank.org/100096

#### Surirella ovalis var. apiculata

Taxon classificationPlantaeSurirellalesSurirellaceae

O. Müll. in Bot. Jahrb. Syst. 34: 36, pl. 2: fig. 10. 1903.

##### Lectotype

(designated by Cocquyt and Jahn 2007c). [icon] Müller (1903): pl. 2: fig. 10 “basin near the hot spring at Utengule, Tanzania”.

= *Surirella ovalis* [var. apiculata] f. minor O. Müll. in Bot. Jahrb. Syst. 34: 36, pl. 2: fig. 11. 1903.

**Lectotype** (designated by Cocquyt and Jahn 2007c). [icon] Müller (1903): pl. 2: fig. 11 “Lake Rukwa”.

#### Surirella pseudotenuis

Taxon classificationPlantaeSurirellalesSurirellaceae

Cholnoky in Portugaliae Acta Biol. Sér. B. 4: 226, fig. 120, 1954.

##### Lectotype

(designated by Cocquyt et al. 2017). UNWH NWU 07–138 “Moss growing on rocks at edge of stram in full sunshine in gully South of road to Vumba, Umtali – 27.7.1952” leg. N.C. Chase.

http://phycobank.org/100097

#### Surirella rudis

Taxon classificationPlantaeSurirellalesSurirellaceae

Hust. in Arch. Hydrobiol. Suppl. 15: 505. 1938.

##### Lectotype

(cited as holotype but in fact designated by Simonsen 1987). BRM X4/3 Lake Tanganyika “Tanganyika See”.

- *Surirella rudis* Hust. in A.W.F. Schmidt, Atlas Diatom.-Kunde, pl. 356: fig. 5, 6. 1922, nom. inval.

#### Surirella sparsipunctata

Taxon classificationPlantaeSurirellalesSurirellaceae

Hust. in Huber-Pestalozzi, Phytoplankt. Süsswass. vol. 2 (2): 516, fig. 631. 1942.

##### Lectotype

(cited as holotype but designated by Simonsen 1987). BRM X4/30 Lake Tanganyika “Tanganikasee. 3rd Tang. Exp., G.S. West”.

- *Surirella sparsipunctata* Hust. in A.W.F. Schmidt, Atlas Diatom.-Kunde, pl. 309: fig. 15. 1914, nom. inval.

= Surirella sparsipuncata var. laevis Hust. in Huber-Pestalozzi, Phytoplankt. Süsswass. vol. 2 (2), 517, fig. 631A. 1942.

**Lectotype** (designated by Simonsen 1987). BRM X4/34 Lake Tanganyika “Tanganikasee 6”.

##### Comment.

For taxonomical results and discussion see Cocquyt and Vyverman (1993).

#### Surirella striolata

Taxon classificationPlantaeSurirellalesSurirellaceae

Hust. in Arch. Hydrobiol. 18: 249. 1927.

##### Lectotype

(designated by Simonsen 1987). BRM 224/92 Lake Tanganyika “Tanganyika See. Grund. 6, 2”.

- *Surirella striolata* Hust. in A.W.F. Schmidt, Atlas Diatom.-Kunde, pl. 356: fig. 7. 1922, nom. inval.

#### Surirella subrugosa

Taxon classificationPlantaeSurirellalesSurirellaceae

Cocquyt & R. Jahn nom. nov.

≡ Cymatopleura solea var. rugosa O. Müll. in Bot. Jahrb. Syst. 34: 23, fig. 3. 1904.
≡ Cymatopleura rugosa (O. Müll.) Cocquyt & R. Jahn in Pl. Ecol. Evol. 147 (3): 416. 2014.

##### Lectotype

(designated in Cocquyt and Jahn 2014). B 40 0040252 [http://herbarium.bgbm.org/object/B400040252] (the valve representing the lectotype was published as fig. 3D–E in Cocquyt and Jahn 2014) “Malawi, Lake Malombe, after discharge of Lake Malawi”.

http://phycobank.org/100098

##### Nomenclatural comment.

The new epithet was chosen because of *Surirella rugosa* Bramb. & P.B. Ham.

### Unrevisable and unrevised taxa

*Surirella acanthophora* Giffen in Beih. Nova Hedwigia 21: 145, pl. 4: figs 92–95. 1966.

Holotype. Giffen collection 30/6 “Fort Hare, Cape Province” South Africa.

Surirella asperrima f. rokuprensis Woodhead & Tweed in Rev. Algol. N. S. 5: 144, fig. 4. 1960, nom. inval.

Locality. “Sierra Leone, Rokupr” (Several localities are cited but no type is indicated McNeill et al. 2012, Art. 40.1).

Comment. Taxon unrevisable according to Cocquyt et al. (2013).

*Surirella capensis* Ehrenb. ex Cocquyt & R. Jahn in Cryptog. Algol. 26: 150. 2005.

Lectotype (designated by Cocquyt and Jahn 2005b). BHUPM 130715 b “Lacus in monte Camdebo Graaf Reinet proximo, Provincia Capensis, Africa Meridionalis”.

- *Surirella capensis* Ehrenb., Mikrogeologie 245, 254. 1854, nom. inval.

Taxonomical comment. Species closely related to *Surirella sparsipuncatata* and *Iconella anassae*. Further studies are needed, including SEM to evaluate its taxonomic position, which is only possible if material from the type locality can be obtained; otherwise unrevisible.

Surirella cuspidata f. constricta Hust. in Explor. Parc Natl. Albert. Mission H. Damas 8: 155, pl. 15: fig. 11. 1949.

Lectotype (cited as holotype but in fact designated by Simonsen 1987). BRM 244/34a DR Congo “Belg. Kongo. 39. Karisimbi-See. +3800 m”.

Comment. Sampling site is located very probably on the Rwandan side of the border with DR Congo.

Taxonomic comment: *S. cuspidata* Hust. in Int. Rev. Hydrobiol. Hydrogr. 42: 156, figs 391–393. 1942, described from Indonesia was transferred to *Stenopterobia cuspidata* (Hust.) Vyverman in Bull. Soc. Bot. Belgique 122: 74. 1989. Further studies are needed to evaluate Hustedt’s forma and its taxonomic position.

Surirella engleri f. densecostata R. Maillard in Bull. Mus. Natl. Hist. Nat. [Paris], Bot. 30: 39, 43 Pl. 3: fig. 1. 1977, nom. inval.

Localities. Mali “Congo et Mozambique” (Several localities are cited but no type is indicated McNeill et al. 2012, Art. 40.1).

Surirella engleri f. sierra-leonensis Woodhead & Tweed in Hydrobiologia 12: 202. 1958, nom. inval.

Locality. Sierra Leone: R. Makoke at Maranda (see Cocquyt et al. 2013).

Surirella fuellebornii var. worthingtonii H.Bachm. in Ber. Schweiz. Bot. Ges. 42: 707, 709, pl. 26: fig. 7, 8. 1933.

Locality. Lake Victoria “Victoria Nyanza”.

Surirella gracilis var. africana Cholnoky in Hydrobiologia 7: 184, fig. 82, 83. 1955.

Syntype localities. Rayton-vlei 30 km E of Pretoria, South Africa, “Tümpelchen“ and “Bächlein” leg. Cholnoky.

Surirella gracilis f. constricta Cholnoky in Hydrobiologia 7: 184. 1955.

Type indicated. 10–12 km N of Rayton, 30–35 km NE of Pretoria, South Africa “Leeufonstein Quellen”

*Surirella ignota* Cholnoky in Nova Hedwigia 2: 118, figs 342, 343. 1960.

Type indicated. “Port Shepstone 362, Kleiner, sickernder Seitenbach des Unzimkulwana-Flusses nahe dem Paddock-Eingange im Oribi Gorge. 22.7.1958.”

*Surirella pseudospinifera* Iltis in Rev. Algol. 10 (4): 334. 1972, nom. inval.

- *Surirella acanthophora* Iltis in Rev. Algol. 10 (2) 174, figs 10–12, pl. 2: fig. 3, 4. 1971, nom. inval. et nom. illeg. [non Giffen 1966].

Localities. Chad, Mali “Mare du 3e barrage à Bol. 13°30'N, 14°43'30"E. Puits près de la mare de Latir. 13°36'N, 14°44'E” (Two localities are cited but no type is indicated McNeill et al. 2012, Art. 40.1).

Comment. *Surirella pseudospinifera* Iltis was the intended substitute name for *Surirella acanthophora* Iltis [non Giffen 1966] but based on an invald name (McNeill et al. 2012, Art. 40.1).

*Stenopterobia recta* Woodhead & Tweed in Hydrobiologia 12: 202, fig. 72. 1958.

Locality. Sierrra Leone, Lake Sofon.

Comment. Taxon unrevisable according to Cocquyt et al. (2013).

*Surirella rokuprensis* Woodhead & Tweed in Rev. Algol. 5: 145, fig. 5. 1960, nom. inval.

Locality. “Sierra Leone, Rokupr” (Several localities are cited but no type is indicated McNeill et al. 2012, Art. 40.1).

Comment. Taxon unrevisable according to Cocquyt et al. (2013).

Surirella rudis var. sierra-leonensis Woodhead & Tweed in Rev. Algol. 5: 146, fig. 9. 1960

Type. Mambolo (2352).

Comment. Taxon unrevisable according to Cocquyt et al. (2013).

*Surirella rudis* [var. sierra-leonensis] f. constricta Woodhead & Tweed in Rev. Algol. 5: 146, fig. 7. 1960, nom. inval.

Locality. Sierra-Leone (Several localities are cited but no type is indicated McNeill et al. 2012, Art. 40.1).

Comment. Taxon unrevisable according to Cocquyt et al. (2013).

*Surirella scutum* Reichelt in A.W.F. Schmidt, Atlas Diatom.-Kunde, pl. 295: fig. 4. 1913, nom. inval.

Locality. Kalahari.

Comment. Type and description of the depicted species are missing.

*Surirella subrobusta* Hust. in A.W.F. Schmidt, Atlas Diatom.-Kunde, pl. 353: fig. 1. 1922.

Lectotype (cited as holotype but in fact designated by Simonsen 1987). BRM 4/59 “Lafirio-Fluß. Deutsch-O-Afrika” (Simonsen 1987).

Comment. Description in the caption of the plate.

*Surirella schweickerdtii* Cholnoky in Bot. Not. 1954: 290, figs 95, 96. 1954.

≡ *Stenopterobia schweickerdtii* (Cholnoky) Brassac, T.Ludwig & Torgan in Diatom Research 18: 186. 2003.

Locality. “Moosrasen auf einer kleinen Insel zwischen Gras. Debegeni” South Africa.

*Surirella taiaensis* J.R. Carter & Denny, Beih. Nova Hedwigia 73: 325, pl. 8: fig. 274. 1982.

Holotype. BM 78108 “Sierra Leone, River Jong (Taia) at Njala”.

Surirella tenera var. minor Cholnoky in Portugaliae Acta Biol. Sér. B, 6: 140 fig. 168. 1958.

Holotype. FR 118 “In rivulo apud Modderpoort prope oppidum Nylstroom (Transvaal)”.

*Surirella welshii* Cholnoky 1962 in Hydrobiologia 20: 337, fig. 45. 1962.

Type indicated. “Unnamed mountain stream between Piggs Peak and Mbabane, 3.7.1961, leg. H. Welsh”, “Swaziland”.

## Conclusion

55 taxa – formerly ranked within *Surirella* - have been transferred to *Iconella*; most of these have been shown to be endemic (Ross 1983, Cocquyt et al. 1993, Cocquyt and Vyverman 1994, Cocquyt 1998, 2000,) and many of them, especially the large species, have become planktonic in the East African great lakes (Müller 1905, Hustedt in Huber-Pestalozzi 1942, Cocquyt 1998). In addition, two taxa – formerly ranked within *Stenopterobia* – have been transferred to *Iconella*. 10 taxa have stayed within *Surirella*, (although the position of *S. sparsipunctata* has to be genetically verified), and six taxa have been transferred from *Cymatopleura* to *Surirella*. For completeness sake, 21 taxa have been listed which are either unrevised or unrevisable because missing morphological data do not allow us to decide if the raphe is raised on a keel.

When more taxa from the genera *Iconella* and *Surirella* have been studied molecularly, especially the endemic species from Africa and other tropical regions, further autapomorphies might be discovered which might support the differentiation into further groups. With the currently available data, the solution by Ruck et al (2016a, b) clarifies their phylogeny and presents a very workable approach.

## Supplementary Material

XML Treatment for Iconella bifrons

XML Treatment for Iconella splendida

XML Treatment for Surirella librile

XML Treatment for Surirella undulata

XML Treatment for Iconella aculeata

XML Treatment for Iconella acuminata

XML Treatment for Iconella anassae

XML Treatment for Iconella africani-orientalis

XML Treatment for Iconella agonaensis

XML Treatment for Iconella approximata

XML Treatment for Iconella bonsaensis

XML Treatment for Iconella brevicostata

XML Treatment for Iconella brevicostata var. constricta

XML Treatment for Iconella brevicostata var. elongata

XML Treatment for Iconella chasei

XML Treatment for Iconella cataractarum

XML Treatment for Iconella chepurnovii

XML Treatment for Iconella coei

XML Treatment for Iconella congolensis

XML Treatment for Iconella crawfordii

XML Treatment for Iconella debesii

XML Treatment for Iconella delicatissima var. ghanaensis

XML Treatment for Iconella dodowaensis

XML Treatment for Iconella dumae

XML Treatment for Iconella ebalensis

XML Treatment for Iconella effusa

XML Treatment for Iconella engleri

XML Treatment for Iconella esamangensis

XML Treatment for Iconella friedelhinziae

XML Treatment for Iconella fuellebornii

XML Treatment for Iconella gradifera

XML Treatment for Iconella heidenii

XML Treatment for Iconella kusberi

XML Treatment for Iconella lancettula

XML Treatment for Iconella latecostata

XML Treatment for Iconella likomensis

XML Treatment for Iconella linearis var. elliptica

XML Treatment for Iconella linearis var. elongata

XML Treatment for Iconella malombae

XML Treatment for Iconella margaritifera

XML Treatment for Iconella margaritacea

XML Treatment for Iconella muelleri

XML Treatment for Iconella murielae

XML Treatment for Iconella nagbogensis

XML Treatment for Iconella nervosa

XML Treatment for Iconella nyassae

XML Treatment for Iconella obtusiuscula

XML Treatment for Iconella oliffii

XML Treatment for Iconella panganiensis

XML Treatment for Iconella plana

XML Treatment for Iconella propinqua

XML Treatment for Iconella pseudothienemannii

XML Treatment for Iconella reicheltii

XML Treatment for Iconella sorriensis

XML Treatment for Iconella spiraloides

XML Treatment for Iconella subcontorta

XML Treatment for Iconella takoradiensis

XML Treatment for Iconella tchadensis

XML Treatment for Iconella tumida

XML Treatment for Iconella turbo

XML Treatment for Iconella vasta

XML Treatment for Iconella vasta var. linearis

XML Treatment for Surirella afrocalcarata

XML Treatment for Surirella clavata

XML Treatment for Surirella comperei

XML Treatment for Surirella distinguenda

XML Treatment for Surirella fasiculata

XML Treatment for Surirella laticeps

XML Treatment for Surirella modesta

XML Treatment for Surirella nyansae

XML Treatment for Surirella olungensis

XML Treatment for Surirella ostentata

XML Treatment for Surirella ovalis var. apiculata

XML Treatment for Surirella pseudotenuis

XML Treatment for Surirella rudis

XML Treatment for Surirella sparsipunctata

XML Treatment for Surirella striolata

XML Treatment for Surirella subrugosa
